# Improving mental health and well-being of hospital staff: mixed-methods process evaluation of the SEEGEN trial

**DOI:** 10.1186/s12889-025-24101-4

**Published:** 2025-08-16

**Authors:** Bernd Puschner, Maja Stiawa, Martin Peters, Peter Angerer, Felicitas Rapp, Florian Junne, Melanie Genrich-Hasken, Andreas Müller, Imad Maatouk, Madeleine Helaß, Nadine Mulfinger, Harald Gündel

**Affiliations:** 1 Department of Psychiatry II, Ulm University, Lindenallee 2, Günzburg, 89312 Germany; 2 Institute of Occupational, Social and Environmental Medicine, Centre for Health and Society, Heinrich-Heine-University, Moorenstraße 5, Düsseldorf, 40225 Germany; 3 Department of Anesthesiology and Intensive Care Medicine, University Hospital Ulm, Albert-Einstein-Allee 23, Ulm, 89081 Germany; 4 Department of Psychosomatic Medicine and Psychotherapy, University Hospital Tübingen, Osianderstraße 5, Tübingen, 72076 Germany; 5 Department of Psychosomatic Medicine and Psychotherapy, Otto von Guericke University Magdeburg, Leipziger Str. 44, Magdeburg, 39120 Germany; 6 Institute of Psychology, Work and Organizational Psychology, University of Duisburg-Essen, Universitätsstraße 2, Essen, 45141 Germany; 7 Department of Internal Medicine II, Section of Psychosomatic Medicine, Psychotherapy and Psychooncology, Julius-Maximilian University Würzburg, Oberdürrbacher Str. 6, Würzburg, 97080 Germany; 8 Department of General Internal Medicine and Psychosomatics, University Hospital Heidelberg, Im Neuenheimer Feld 410, Heidelberg, 69120 Germany; 9 Clinic of Psychosomatic Medicine and Psychotherapy, University Hospital Ulm, Albert-Einstein-Allee 23, Ulm, 89081 Germany

**Keywords:** Mental health, Hospital, Work-related stress, Cluster-Randomised controlled trial, Process evaluation, Well-Being

## Abstract

**Background:**

Health workers experience high levels of occupational stress. There is a lack of knowledge about moderators and active ingredients of interventions aiming to reduce occupational stress in health workers. This paper presents results of the mixed-methods process evaluation of the SEEGEN (“SEElische GEsundheit am Arbeitsplatz KrankeNhaus) randomised controlled trial.

**Methods:**

Employees of three hospitals in Germany were recruited for this trial between 10/2019 and 01/2020. Participants allocated to the intervention group could choose from five modules according to their needs and interests. Outcome and process measures were collected with established scales, including emotional and cognitive strain (Irritation Scale), subjective well-being, psychosocial safety climate, and self-efficacy. Uptake of the intervention and fidelity was assessed using the SEEGEN Fidelity Scale. Multivariate general least squares ANOVA with allocation as interaction effect was used for analysis of moderators. Mediator analysis was done using ordinary least square regression. Qualitative data was collected via semi-structured interviews from a sub-sample, focusing on organisation, outcomes and practical applicability of the intervention. Interviews were analysed using qualitative content analysis.

**Results:**

415 hospital employees participated in this study. Average dose of the intervention across all modules was a close to two days. The effect of the intervention was moderated by some staff- and work-related variables, and mediated by fidelity to the intervention. Qualitative results of 23 participants confirmed and supplemented quantitative findings. The participants’ adjustments in terms of stress reduction related primarily to the individual level, in particular to changes in behaviour and the altered perception of work situations. Cross-study (data type) synthesis enabled a deeper understanding of the moderating variables of the intervention with regard to age of participants and the relevance of contextual conditions and confirmed the range of requirements of the participants depending on their respective work situation.

**Conclusions:**

The high complexity of the intervention with different modules and target groups hindered implementation and effectiveness. A step backwards seems indicated, i.e. the further evaluation of smaller components of the intervention, added by theory development and refinement.

**Trail registration:**

DKRS, DRKS00017249. Registered 08/10/2019, https://www.drks.de/DRKS00017249.

**Supplementary Information:**

The online version contains supplementary material available at 10.1186/s12889-025-24101-4.

## Contributions to the literature

- We investigated what contributes to the usefulness of a complex intervention to reduce stress from the perspective of hospital workers.
- We found that study participants appreciated the intervention, but they also reported that it was too short and rather complicated, and hardly addressed the context of their work environment which was seen as the main reason for stress.
- Our findings shed light on the importance of contextual variables when investigating complex interventions.
- Based on this work, pertinent parts of the intervention may be further developed, implemented, and tested.

## Background

Health workers experience high levels of occupational stress, with potential deleterious effects on their mental health [1–3]. The COVID-19 pandemic has placed an additional heavy toll on already exhausted health workers [4]. Systematic reviews have summarised the evidence-base for a considerable number of individual- or organisation-directed interventions to reduce or prevent occupational stress in health workers, showing overall small to medium effects and low study quality (e.g. low sample sizes, paucity of medium- or long-term data) [5, 6]. The high variation of effect sizes across studies implies a that a number of mediating or moderating variables affect the effectiveness of such interventions [7, 8]. The field should pay more attention to contextual and process issues [9].

Analysis of mediators and moderators is the very task of process evaluation, in order to better understand why, under which conditions, and for whom an intervention works or does not work. Process evaluations investigate programme fidelity, quality of implementation, contextual factors, and mechanisms of impact, to better understand the role of components of complex interventions and their contributions to variations in outcomes [10–13]. Process evaluations using mixed-methods yield rich contextual knowledge which is much needed, especially when evaluating psychosocial interventions [14, 15]. Thus, recent state-of-the-art guidance for evaluating complex interventions in healthcare and public health settings has also recommended to prioritise mixed-methods approaches which are more sensitive to complexity and emphasise context and system fit [16].

There is great heterogeneity of variables used in process evaluations of organisational stress management interventions [8, 17]. Nonetheless, it has been convincingly demonstrated that self-efficacy is an important mediator between job stress and mental health among health workers [18–22], and should therefore be included in process evaluations. Another vital element of process evaluation is fidelity which refers to the degree to which an intervention is delivered as intended [23, 24]. Measuring fidelity usually includes a number of elements, such as adherence to an intervention, exposure or dose, quality of delivery, participant responsiveness, and programme differentiation [25]. Further, some reviews summarised the evidence on barriers and facilitators to implementation of interventions in hospitals. For example, based on 43 mostly qualitative studies, Geerligs and colleagues found bi-directional associations between three domains of barriers and facilitators to implementation (system, staff, and intervention) [26]. Moreover, Cowie and colleagues, based on 32 also mostly qualitative studies, identified a transparent clear identification of responsible persons, backing of the intervention by decision-makers, and sufficient organisational support as key facilitators supporting sustainable implementation. In contrast, insufficient staffing level and, as a consequence, insufficient time to acquire and engage with intervention mechanisms were identified as the most outstanding barriers [27].

Over the last years, some process evaluations of occupational stress management programmes have been put forth [28]. From the beginning, the field has struggled with incomplete reporting of information relevant to process evaluation, and great variety in methods, making it difficult to identify reliable determinants of effective intervention implementation or outcomes, and impeding progress of process evaluation theory building [9, 17, 28]. Mixed-methods process evaluations alongside RCTs of complex inventions to improve the well-being of health workers are rare. We could only identify one such study which examined fidelity in a trial of a complex team-based behavioural intervention delivered to teams in nursing homes [29]. It was found that the mere dose was less important than the intensity with which intervention participants enacted the core components of the intervention.

To date, no mixed-method process evaluation of a complex intervention to reduce occupational stress in hospital employees has been conducted in Germany. Thus, this study provides a mixed-methods process evaluation of the SEEGEN trial which tested the effects of a complex intervention on primary (work stress) or secondary outcomes (well-being and psychosocial safety climate) [30]. The quantitative part of this process evaluation will specify moderators and mediators of the effect. In addition, qualitative methods will be used to explore participants’ attitudes, perceptions, and experiences with the intervention, as well as factors facilitating or hindering implementation. Finally, results of both quantitative and qualitative analyses will be combined in a cross-study (data type) synthesis [31].

## Methods

### Design

This study is a mixed-methods process evaluation of the multi-centre cluster-randomised controlled trial “Mental health in the hospital workplace” (SEElische GEsundheit am Arbeitsplatz KrankeNhaus – SEEGEN; ID DRKS00017249). The study including this process evaluation was approved by the ethics committees of the participating study centres (Ulm University: 501/18, Heidelberg University: S-602/2019, Düsseldorf University: 6193R). The process evaluation was preplanned to be carried out regardless of the results of the RCT, i.e. whether a (strong) effect could be established or not [32]. Results of this process evaluation are reported adhering to relevant guidance [33, 34]. Recruitment and baseline assessment (T0) took place from 10/2019 to 01/2020 at three hospitals (A: owned by a private health company; B: community hospital; C: university hospital), followed by post intervention assessment (T1, 6 months), and follow-up (T2, 12 months). Inclusion criteria were 18–70 years of age, having provided written informed consent, and sufficient command of the German language. Participants allocated to the intervention group could choose from five modules according to their specific needs and interests depending on their position in the hospital: (1) Top Management Training, (2) Promoting Stress Preventive Relational Leadership Competence, (3) Dilemma Competency – Coping by Taking Responsibility, (4) Reconciling Work and Family Life and (5) Staying Healthy at Work. See the study protocol for further details on design, recruitment, randomisation, and intervention of the SEEGEN trial [35].

### Quantitative component of the process evaluation

#### Measures

Data used in this part of the process evaluation from all participants included information on socioeconomic status, work situation and history, outcome and process measures, uptake of the intervention, and fidelity.

Outcome measures included the primary outcome (Irritation Scale, IRR [36]), which measures emotional and cognitive strain in the work context using 8 items on a 7-point Likert scale (1 “not at all” to 7 “almost completely correct”), with the IRR total score representing the sum of all items, with higher values reflecting higher strain. Furthermore, subjective well-being was measured with the World Health Organization Well-Being Index (WHO-5 [37]) with 5 items on a 6-point Likert scale (0 “at no time” to 5 “all of the time”), whose sum was multiplied by 4 to give the final score ranging from 0 representing the worst well-being to 100 representing the best well-being. Additionally, psychosocial safety climate was measured using the Psychosocial Safety Climate Scale (PSC-12 [38]) with 12 items on a 5-point Likert scale (1 “strongly disagree” to 5 “strongly agree”), with the PSC-12 total score representing the sum of all items, higher values reflecting higher psychosocial safety. Process measure was the short version of the Occupational Self-Efficacy Scale (SOSES [37]) which consists of 6 items rated on a 6-point Likert scale (1 “not at all true” to “6 “completely true”), with higher sum score values reflecting higher occupational self-efficacy.

Uptake of the intervention and fidelity was assessed using the SEEGEN Fidelity Scale which was developed between June and November 2019 in collaboration with representatives of study teams of all sites following current guidelines [39, 40]. After intensive literature search and scrutiny of existing measures and recommendations [41–43], consensus was reached that the scale should focus on general operational fidelity (exposure, engagement, enactment), trainer competency, and overall satisfaction with the intervention, and that mode of application should be self-report. The initial item pool consisted of 29 items, of which 10 were excluded due to redundancy and lack of relevance or face validity. Two of the remaining 19 items measured uptake of to each of the five intervention modules, and reasons for participation. Dose was the number of days a participant attended any of the workshops. Further items, each assessed on 5-point Likert scales (0 “not true”; 1 “hardly true”; 2 “partly true”; 2 “rather true”; 4 “absolutely true”), measured individual engagement, i.e. the extent to which participants were involved in the intervention (4 items, #1–4); enactment, i.e. the extent to which the participants implemented the activities from the workshop and the recommendations learned in their daily lives (4 items, #5–8), competence and skills of the trainer and how the quality of implementation and the interpersonal and process skills were perceived (4 items, #9–12); and general satisfaction with the intervention (5 items, #13–17). The SEEGEN Fidelity Scale total score was the mean over all 17 items. See Table S1 in the online supplement for the complete SEEGEN Fidelity Scale. Stress due to the pandemic was measured at T1 with one item (“How stressed have you felt in the last 2 weeks, including today, due to the COVID-19 pandemic?”), rated on a 5-point Likert scales (1 “not at all” to 5 “very much”).

#### Analysis

Descriptive reports include absolute and relative frequencies for categorical variables, and means and standard deviation for continuous variables. All scales’ total scores were calculated on the condition that at least 80% of items part of the scale were completed, and sum scores prorated as needed. Internal consistency of the SEEGEN Fidelity Scale was established by calculating Cronbach’s alpha, and its change over time was analysed using a paired t-test. Multivariate general least squares ANOVA with allocation as interaction effect was used for analysis of moderators. In line with recent recommendations, mediator analysis followed a simple model [44], using ordinary least square regression. For both moderator and mediator analysis, outcome and process measures were converted to standardised residual (change) scores, taking into account previous change by partialing out (t1 scores were controlled for change between t0 and t1). All analyses were carried out with SPSS version 27.

#### Qualitative component of the process evaluation

Study participants allocated to the intervention group were invited for an additional interview. Participants were interviewed about their experiences of participating in the intervention using a semi-structured interview-guide tapping into their experiences with organisational, content-related and application-oriented aspects of the workshop(s) attended (see Table S2). All participants received detailed written information and gave informed consent. Interviews were held in the fall of 2020 by phone by two researchers with expertise in qualitative research (MS, MP), who were not involved in conducting the intervention workshops. All participants received a book voucher containing 30 Euro. All interviews were audio-recorded and transcribed verbatim in its original language. All transcripts were checked for accuracy by a student research assistant.

Content analysis was conducted in line with Kuckartz [45]. First, all transcripts were carefully read, annotated, paraphrased and summarised in a case-summary by a researcher (MS). Afterwards, all transcripts and case-summaries were reread independently by a second researcher (MP) for additional comments. In the following, aspects which had turned out to be unclear or divergent opinions on transcripts were discussed until joint consensus was achieved. Afterwards, the material was analysed using MAXQDA 20 software. In the following process of analysis, categories were further refined (Table S3).

During the development of the coding system, transcripts were coded independently by two researchers and regularly discussed (MS, MP) until joint consensus was achieved. Topics were coded (and counted) by sentences. If participants’ reporting to a theme exceeded one sentence, the whole section was coded once. If themes were assigned to a subcode, no double coding with the subordinate code was performed. Apart from that, themes could have been assigned to several subcodes within one subordinate code and to other codes in general. A first system of categories was developed by two researchers (MS, MP) on the basis of deductive codes resulting from the interview guide and inductive codes resulting from the initial revision, which was presented and discussed in a qualitative research workshop, and then refined and finalised.

#### Integration of quantitative and qualitative components

According to a convergent mixed-methods design [46], quantitative and qualitative data were collected and analysed at the same time. Initially, quantitative and qualitative data were analysed separately. Afterwards, results were compared with regard to similarities and differences between both types of data was conducted, to deepen our understanding of moderators and mediators of effect, and of the process of implementation of the intervention.

## Results

### Quantitative

#### Participants

Most participants were female, over 50 years old, married, and working full-time as nurses. See Table 1 for details of participant characteristics.

**Table 1:** Participant Characteristics

		IG *n* (%)	CG *n* (%)
Gender	female	139 (33.5)	159 (38.3)
	male or diverse	60 (14.5)	57 (13.7)
Age (years)	21–30	36 (9.3)	34 (8.7)
	31–40	35 (9.0)	55 (14.1)
	41–50	46 (11.8)	45 (11.6)
	50+	69 (17.7)	69 (17.7)
Marital status	married or in partnership	135 (34.3)	153 (38.8)
	single or divorced	54 (13.7)	52 (13.2)
Hospital	A	54 (13.0)	52 (12.5)
	B	83 (20.0)	95 (22.9)
	C	62 (14.9)	69 (16.6)
Working group	medical service	52 (13.3)	57 (14.6)
	nursing service	94 (24.1)	96 (24.6)
	functioning service or other	42 (10.8)	49 (12.6)
Current working situation	full time	124 (31.2)	137 (34.4)
	part time	67 (16.8)	70 (17.6)
Shift work	no	88 (22.2)	91 (23.0)
	yes	102 (25.8)	115 (29.0)
Job experience (years)	0–15	67 (17.5)	65 (17.0)
	16–30	53 (13.9)	79 (20.7)
	30+	62 (16.2)	56 (14.7)
Working at current hospital (years)	0–10	70 (18.4)	86 (22.6)
	11–20	42 (11.0)	46 (12.1)
	21+	70 (18.4)	67 (17.6)
Leadership/management responsibility	no	132 (31.8)	117 (28.2)
	yes	67 (16.1)	99 (23.9)
*n*=415; IG (intervention group): *n*=199 (48.0%); CG (control group): *n*=216 (52.0%)			

#### Uptake of the intervention and fidelity

Most participants who took part in the intervention modules #2 (Leadership Competence), #3 (Dilemma Competence), or #5 (Staying Healthy), while modules #1 (Management) and #4 (Work and Family) were attended less often. Average dose of the intervention across all modules was a bit less than two days at each measurement point, with a total of two and a half days across the entire period of delivery of the intervention. Participation was primarily motivated by the hope to get ideas for everyday work, followed by personal growth, and hardly by recommendations from employers or colleagues. See Table 2 for details.

**Table 2:** Uptake of the intervention, reasons for participation, and fidelity

	t1	t2
Uptake		
1. Top management training (1 day^a^), *n (%)*	7 (7.4)	2 (2.9)
2. Stress preventative leadership (2 days), *n (%)*	25 (26.6)	11 (15.9)
3. Dilemma competence (2 days), *n (%)*	25 (26.6)	25 (36.2)
4. Work and family (1 day), *n (%)*	19 (20.2)	8 (11.6)
5. Staying healthy (2 days), *n (%)*	23 (24.5)	24 (34.7)
Total days, *mean (sd)*,* min-max*	1.83 (0.6), 1–4	1.88 (0.4), 1–4
Reasons for participation		
To get ideas for everyday work, *n (%)*	78 (83.0)	57 (83.8)
Personal growth, *n (%)*	51 (54.3)	38 (55.9)
Recommendation of employer, *n (%)*	9 (9.6)	6 (8.8)
Recommendation of colleagues, *n (%)*	3 (3.2)	1 (1.5)
Fidelity		
Fidelity total, *mean (sd)*	3.03 (0.69), *N* = 73	3.05 (0.59), *N* = 57
Fidelity, cronbach’s alpha	α = 0.94, *N* = 65	α = 0.91, *N* = 49

^a^Equalling one working day, i.e. appr. 8 h excluding breaks. N at t1 = 94 (missing *N* = 105); N at t2 = 69 (missing *N* = 130); Total days (mean (sd), min-max): 2.46 (1.13), 1–8. Change fidelity t1-t2: T_paired_= 1.21; df = 26; *p* = 0.23

As also shown in Table 2, internal consistency of the 17-item SEEGEN fidelity scale was high at both t1 and t2, and did not change over time.

#### Moderation

**Fig. 1:**
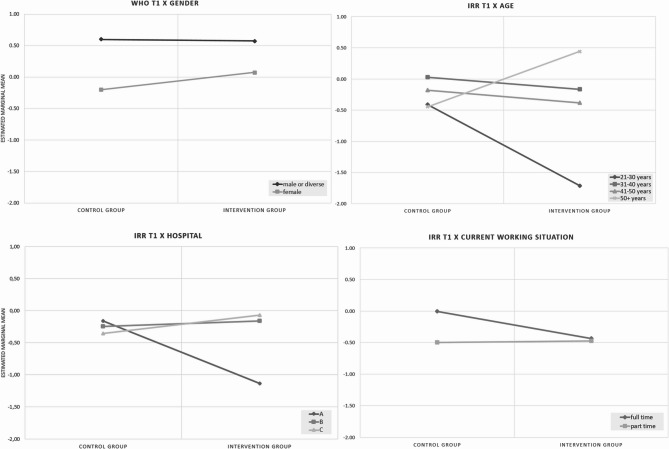
Moderators of effect

Descriptives of outcome and process measures are shown in Table S4, showing that all measures remained stable over time regardless of allocation. Multivariate GLM analysis yielded 8 (of 60 possible) moderator effects, i.e. the effect of the intervention differed between intervention and control groups for gender (for WHO-5 at t1), age (for IRR at t1), marital status (for IRR and WHO-5 at t2), study site (for IRR and WHO-5 at t1), duration of current employment (for IRR at t1), and leadership role (PSC-12 at t1) (Table 3). Selected effects are illustrated in Fig. 1, showing that (i) WHO-5 at t1 in females allocated to the intervention group improved (vs. control group), as compared to no difference in males; (ii) IRR at t1 in younger participants allocated to the intervention group decreased (vs. control group), while it increased in older participants, and remained stable among 30–50 year olds; (iii) IRR at t1 in participants at hospital A allocated to the intervention group reduced (vs. control group), while it remained stable at the other two study sites; and that (iv) IRR at t1 in participants who worked full time allocated to the intervention group reduced more (vs. control group), as compared to participants working part-time.

**Table 3:** Moderators (interaction effects by allocation)

Source	Dependent variable	Type III sum of squares	df	Mean of squares	F	*p*
Gender	IRR t1	2.855	2	1.427	1.771	0.174
	WHO-5 t1	12.034	2	6.017	6.474	0.002
	PSC t1	1.583	2	0.791	0.910	0.405
	IRR t2	1.515	2	0.758	0.735	0.481
	WHO-5 t2	0.486	2	0.243	0.257	0.774
	PSC t2	0.588	2	0.294	0.291	0.748
Age	IRR t1	15.728	6	2.621	3.253	0.005
	WHO-5 t1	6.132	6	1.022	1.100	0.365
	PSC t1	5.056	6	0.843	0.969	0.448
	IRR t2	5.931	6	0.988	0.959	0.455
	WHO-5 t2	5.599	6	0.933	0.986	0.437
	PSC t2	6.710	6	1.118	1.105	0.362
Marital status	IRR t1	2.688	2	1.344	1.668	0.192
	WHO-5 t1	3.501	2	1.750	1.883	0.156
	PSC t1	2.383	2	1.191	1.370	0.257
	IRR t2	7.093	2	3.547	3.440	0.035
	WHO t2	8.829	2	4.415	4.666	0.011
	PSC t2	0.882	2	0.441	0.436	0.648
Hospital	IRR t1	11.525	4	2.881	3.575	0.008
	WHO-5 t1	10.172	4	2.543	2.736	0.031
	PSC t1	7.182	4	1.795	2.065	0.088
	IRR t2	7.174	4	1.793	1.740	0.144
	WHO-5 t2	9.216	4	2.304	2.435	0.050
	PSC t2	1.828	4	0.457	0.451	0.771
Working group	IRR t1	2.298	4	0.575	0.713	0.584
	WHO-5 t1	4.608	4	1.152	1.240	0.297
	PSC t1	5.411	4	1.353	1.556	0.189
	IRR t2	3.785	4	0.946	0.918	0.455
	WHO-5 t2	1.378	4	0.344	0.364	0.834
	PSC t2	4.610	4	1.153	1.138	0.341
Current working situation	IRR t1	4.083	2	2.041	2.533	0.083
	WHO-5 t1	3.350	2	1.675	1.802	0.168
	PSC t1	0.040	2	0.020	0.023	0.977
	IRR t2	0.886	2	0.443	0.430	0.652
	WHO-5 t2	4.067	2	2.033	2.149	0.120
	PSC t2	2.378	2	1.189	1.174	0.312
Shift work	IRR t1	2.337	2	1.168	1.450	0.238
	WHO-5 t1	0.633	2	0.316	0.340	0.712
	PSC t1	0.979	2	0.489	0.563	0.571
	IRR t2	0.476	2	0.238	0.231	0.794
	WHO-5 t2	4.165	2	2.083	2.201	0.114
	PSC t2	0.623	2	0.312	0.308	0.735
Job experience	IRR t1	2.173	4	0.543	0.674	0.611
	WHO-5 t1	3.605	4	0.901	0.970	0.426
	PSC t1	2.422	4	0.605	0.696	0.596
	IRR t2	3.770	4	0.942	0.914	0.457
	WHO-5 t2	7.128	4	1.782	1.884	0.116
	PSC t2	2.535	4	0.634	0.626	0.645
Working at current hospital	IRR t1	13.679	4	3.420	4.244	0.003
	WHO-5 t1	2.662	4	0.665	0.716	0.582
	PSC t1	4.807	4	1.202	1.382	0.243
	IRR t2	2.597	4	0.649	0.630	0.642
	WHO-5 t2	3.014	4	0.753	0.796	0.529
	PSC t2	1.989	4	0.497	0.491	0.742
Leadership	IRR t1	3.239	2	1.620	2.010	0.138
	WHO-5 t1	1.642	2	0.821	0.883	0.416
	PSC t1	9.608	2	4.804	5.526	0.005
	IRR t2	1.861	2	0.931	0.903	0.408
	WHO-5 t2	3.138	2	1.569	1.659	0.194
	PSC t2	0.080	2	0.040	0.039	0.961

Multivariate generalised linear model, *N* = 188, only complete cases analysed

#### Mediation

Meditation analyses showed that fidelity affected the effect of the intervention for two outcomes (IRR and WHO-5) at t1 only. Effect of the intervention was independent of other variables (dose, SOSES) at t1, and in addition of previous change in SOSES and previous stress due to COVID-19 at t2 (Table 4).

**Table 4:** Mediation

Time	Scale	Coefficient	β	CI lower	95% upper	SE	T	*p*
T1	IRR (*N* = 68)	constant	1.487	− 0.351	3.325	0.920	1.616	0.111
		Dose (days) t1	− 0.017	− 0.458	0.425	0.221	− 0.076	0.940
		Fidelity total t1	− 0.540	− 0.958	− 0.121	0.209	−2.578	0.012
		SOSES t0	0.052	− 0.231	0.334	0.141	0.365	0.716
	WHO-5 (*N* = 68)	constant	−1.732	−3.441	− 0.023	0.855	−2.025	0.047
		Dose (days) t1	− 0.067	− 0.478	0.343	0.205	− 0.328	0.744
		Fidelity total t1	0.719	0.330	1.108	0.195	3.693	< 0.001
		SOSES t0	− 0.061	− 0.324	0.201	0.131	− 0.467	0.642
	PSC (*N* = 66)	constant	− 0.974	−2.799	0.851	0.913	−1.067	0.290
		Dose (days) t1	0.116	− 0.322	0.554	0.219	0.530	0.598
		Fidelity total t1	0.163	− 0.253	0.578	0.208	0.783	0.437
		SOSES t0	0.034	− 0.246	0.314	0.140	0.241	0.810
T2	IRR (*N* = 39)	constant	1.179	− 0.648	3.006	0.902	1.307	0.199
		Dose (days) total	− 0.047	− 0.305	0.212	0.128	− 0.364	0.718
		Fidelity total t2	− 0.343	− 0.842	0.156	0.246	−1.392	0.172
		SOSES change t0-t1	− 0.128	− 0.395	0.140	0.132	− 0.965	0.341
		Covid stress t1	− 0.031	− 0.291	0.229	0.128	− 0.241	0.811
	WHO-5 (*N* = 39)	constant	0.544	−1.336	2.424	0.928	0.586	0.561
		Dose (days) total	− 0.117	− 0.383	0.149	0.131	− 0.889	0.380
		Fidelity total t2	0.008	− 0.505	0.522	0.253	0.033	0.974
		SOSES change t0-t1	0.047	− 0.228	0.323	0.136	0.349	0.729
		Covid stress t1	− 0.076	− 0.344	0.191	0.132	− 0.578	0.567
	PSC (*N* = 40)	constant	− 0.168	−2.194	1.858	1.001	− 0.168	0.867
		Dose (days) total	− 0.041	− 0.328	0.246	0.142	− 0.287	0.776
		Fidelity total t2	0.186	− 0.367	0.739	0.273	0.681	0.500
		SOSES change t0-t1	− 0.022	− 0.319	0.275	0.147	− 0.149	0.882
		Covid stress t1	− 0.065	− 0.353	0.223	0.142	− 0.460	0.648

Only complete cases analysed. Analyses with missings replaced (by mean) yielded similar results, i.e. same two mediators (*p* < 0.05)

### Qualitative

The 23 participants in the qualitative interviews were employees of all study sites, and each intervention workshop was attended by at least one participant. Most participants were female, and participants represented a broad spectrum of professional groups (physicians, nurses, administration, management) and different hierarchy levels. Each participant attended at least one of the various workshops, ensuring that participants’ feedback reflected their experience of all the workshops delivered as part of the intervention. Most workshops attended were held face-to-face, but a few were held online because of restrictions due to the COVID-19-pandemic. Four participants were unable to visit a workshop, but remained in the sample to also obtain information about barriers to participation (Table 5).

**Table 5:** Participant characteristics qualitative interviews (*n* = 23)

		*N* (%)
Gender	Female	14 (61)
	Male or diverse	9 (39)
Professional group	Physician	9 (39)
	No managerial responsibilities	3 (13)
	Lower/intermediate management	3 (13)
	Senior management	2 (9)
	Nurse/dental nurse	13 (57)
	No managerial responsibilities	11 (48)
	Lower/intermediate management	2 (9)
	Senior management	-
	Administration/Management	1 (4)
	No managerial responsibilities	-
	Lower/intermediate management	-
	Senior management	1 (4)
Participation	Online	2 (9)
	In person	17 (74)
	No participation	4 (17)

The duration of the interviews with workshop participants varied between 22 and 53 min, with a mean of 32 min. The interviews with participants who did not attend any workshop lasted between 7 and 17 min, with a mean of 13 min. The results of the analysis contained four themes and sixty subcategories (table S3).

In the following, results will be reported along the themes (a) project support/health promotion, (b) organisational aspects, (c) content-related aspects of the workshops, and (d) implementation and knowledge transfer.

#### Project support / health promotion

Contextual factors must be considered fundamental when describing the process of intervention implementation, as they affect the intervention mechanisms, outcomes and implementation processes. In this context, participants described how they perceived the overall importance of occupational healthcare and mental health promotion in their hospital workplace and how they experienced employer support for the project, as well as barriers and facilitators to participation in the workshop.

All participating hospital sites supported the project by providing the necessary infrastructure such as meeting rooms. They further allowed for short project presentations within their team meetings. Hospital staff were given the opportunity to take part in a workshop during their working hours. Participants’ experiences regarding their actual opportunity to participate varied. Participants who work half-time or during night shifts in particular reported that participation in the workshops was easily compatible with their working time model. From the point of view of some participants, support of the intervention from management and superiors is an essential prerequisite for creating the opportunity to participate in the intervention at their workplace.

In contrast, other participants reported a number of barriers to participation, some of which were due to hospital-specific working conditions and structural circumstances. In this regard, some participants reported that they were not allowed to participate in order to meet the work schedule or that the originally approved participation was withdrawn due to staff shortages in order to ensure patient care. As a result, some participants reported that they attended workshops only partially, not at all or in a smaller number than they had initially planned:

*»So*,* I was disappointed*,* but I accepted it*,* we were just not enough people*,* too many people have been ill. What was I supposed to do? Of course*,* I work. That applies to other trainings as well. If there are only a few people*,* you have to cancel the training.« (Nurse)*

Further, some participants described that they felt project support and encouragement of hospital staff to participate was sporadic and not consistently promoted at management level. As one physician put it, the hospital site could have gained more from the intervention through greater management commitment:

*»There was goodwill*,* but in the end*,* it was not given high priority. So*,* one took note of it well-disposed and said*,* yes*,* we will be part of it*,* but they could have also said*,* it [the project; M.S.] is important to us and we want avail oneself from this opportunity and they did not do it that way.« (Physician)*

Many participants of all professional fields and professional hierarchies discussed the great importance of corporate healthcare and, in particular, mental health promotion against the background of what they consider to be the rather low priority of such services in their workplace. Accordingly, participants described what they perceived to be inadequate project support in their hospital as symptomatic of a lack of sustainability with relation to such services and mental health promotion in particular:

*»Like*,* yes*,* mental health*,* we should take care of our employees*,* so let´s provide something. But sometimes I miss the seriousness (…). Sometimes I would like to feel that it is really important to them. Sometimes you get the impression that*,* well*,* maybe they just want to show that they have done something without really supporting it.« (Physician)*

Participants considered several reasons for the in their view low priority of occupational healthcare and mental health promotion at the management level. While some participants described their impression that »*… the idea*,* that you have to take good care of your employees to keep them*,* is not very strong. « (Nurse)*, others described their impression that administrative staff are overburdened due to excessive demands which is why the capacity for corporate healthcare is limited:

*»And*,* as I said*,* I am not assuming malice*,* but simply that their duties are overwhelming*,* that they cannot cope.« (Nurse)*

In the view of some participants, the Covid-19 pandemic served as a reinforcing factor by absorbing personnel resources and thus further reducing the administrative capacity for general occupational healthcare.

#### Organisational aspects

Participants reported that organisational aspects to realise the workshops have been managed successfully by project staff and were correspondingly adjusted to their workplace and working conditions in general. Against the background of participants working conditions, the following organisational aspects turned out as relevant factors in terms of workshop participation: the place of event, project staff reachability and reference persons, the nature and extent of information, as well as the number and arrangement of workshop participants.

##### Venue easy to reach

Due to narrow time frames, a few participants found it convenient that the place of event was located within their place of employment:

*»Especially for me as a mother*,* who has to try and find time to be involved.« (Nurse)*

##### Project staff reachability

Communication between project staff and participants took place primarily via e-mail. Participants appreciated the fast e-mail response and the possibility to ask questions via e-mail. However, one participant reported that she found the changing responsibilities of administrative project staff confusing:

*»Everyone knew what the other one was doing. But I never understood why another person would contact me. So*,* I found it a bit unusual.« (Medical laboratory assistant)*

##### Nature and extent of information

Hospital staff received project information at their workplace by short project presentations given by project staff during team meetings in the hospital departments, by e-mail and through project flyers distributed in the hospital departments. Results showed that participants considered the nature and extent of information as appropriate and assessed the presenting project staff as knowledgeable. One participant appreciated the personal appearance of the project manager:

*»He even came in person and gave a little report. So*,* I thought that was a great commitment in that respect.« (Physician)*

##### The number and arrangement of workshop participants

Many participants emphasized the importance of the number and arrangement of workshop participants. Most of the workshops were eligible for staff members of different professional fields, hospital departments and professional hierarchies. Some participants described the exchange of job experiences between different kinds of staff members as highly rewarding. In the view of participants, such kind of experience exchange raised mutual awareness and profound comprehension of needs between hospital staff:

*»It was some kind of wake-up call in terms of dealing with each other […] because you get to know each other’s problems and then mutual understanding is raised […].« (Medical-laboratory assistant)*

As a consequence, such kind of experience exchange might result in improved cooperation of staff, as one physician exemplified:

*»I myself noticed that - because I got to know the perspective of other professional groups during the workshops–– I was more sensitive to their needs and problems in communication and task sharing such as with my colleagues from the nursing staff.« (Physician)*

##### Workshop modifications resulted from Covid-19

Due to the Covid-19 pandemic, the project staff had to realise several workshop modifications to continue the intervention implementation at hospital sites. Because of the nationwide hospital visiting ban, it was not possible to conduct workshops on site temporarily. To ensure the continuation of the implementation of the intervention, staff members instead obtained the opportunity to take part online. To realise the online workshops, its structure and content needed to be adjusted and timetables needed to be revised by project staff. As a result of the workshop modifications, the duration of workshops sessions was reduced from two days to some hours and the number of participants for a single workshop session was reduced while the number of workshop sessions was scaled up.

Many participants referred to implications of Covid-19 when describing organisational aspects. Participant’s experiences with- and perceptions of workshop modifications varied. Some participants described that they had no difficulties with the adjustments of workshops:

*»We just received the files previously. And then we frequently met and talked via webcam and developed work packages together*,* and further worked out homework assignments for the time between the workshop appointments.« (Physician)*

In contrast, other participants described difficulties to take part, due to technical or organisational reasons. One nurse described that she refused to participate online due to her older age, which probably caused her discomfort with online participation:

*» I wanted to take part at the second one* (Workshop, M.S.) *as well*,* (…) but it would have been an online event*,* and as I belong to the older generation (laughs)*,* I let myself out* (from the session, M.S.).*« (Nurse)*

Some participants described that was difficult to organize participation for organisational reasons. One physician described the problem of changing appointment at short notice against the background of hospital specific working conditions and ensuring patient care:

*»Because in the meantime others had applied for vacation or something else. It was tricky for the employer to say*,* well*,* actually we do not have the capacities to exempt anyone that day*,* whether vacation*,* research or training. And it was somewhat more difficult to assert oneself and to say*,* well*,* officially I already had the permission to take part.« (Physician)*

One nurse described the condensed workshop format as inadequate. In her opinion, this format was not appropriate for adequately addressing contextual issues:

*»And finally*,* it (the workshop*,* M.S.) was just three online sessions lasting one hour each and I found that was falling short. That was not at all comparable anymore in my opinion.« (Nurse)*

#### Workshops– content-related aspects

Participants gave a mixed assessment about how much they considered workshop content appropriate to reduce work related stress in hospital staff. Participants particularly appreciated that they were able to contribute their own personal experiences with challenging work situations, the many practical exercises, the information on possible underlying mechanisms of stress in the hospital workplace and the opportunity to learn about possible individual courses of action with the aim of reducing stress, such as meditation or other possible stress reduction techniques:

*»Potential causes of stress were explained from a professional point of view*,* something you do not really think about as a layman. You can feel it [the stress*,* M.S.]*,* you cannot really avoid it [the stress*,* M.S.] and then someone explained what are potential stressors*,* where does that all comes from and how can I influence it? What can I not influence*,* what can I influence and do by myself? And those things were very*,* very important.« (Nurse)*

In contrast, some participants assessed workshop matters as less helpful to reduce work-related stress among hospital staff, if recommendations for action include or refer to hospital structures. For instance, the participants did not consider possible strategies for organizing breaks to be applicable due to the special working conditions in a hospital, as one physician described:

*»[…] the break*,* because that rarely works in a medical profession. So*,* taking a break for half an hour*,* turning off my phone*,* not being available for anyone*,* that does not exist*,* at least in our department*,* it is not possible. We could not take responsibility for it*, [this suggested solution, M.S.] *was very unhelpful.« (Physician)*

Some participants reported that they had prior knowledge of the topics covered in the workshop. For instance, a few participants referred back to their own long-standing work experience in a leading function. Others mentioned having basic knowledge about mindfulness practises and relaxation techniques and tried to implement these techniques into their work processes on occasion. Nevertheless, these participants could find some food for thought to benefit from the workshop in this way:

*»I did yoga myself for many years*,* something that was actually offered by the hospital and it is really extremely helpful to calm your nerves from time to time. And it really works*,* the word body scan I even did not know and that is something really helpful if you visualize it.«*

#### Implementation and knowledge transfer

When asked to what extent the workshops contributed to coping with- and reducing work-related stress, participants reported various behaviour changes at an individual level, such as a changed perception and handling of (stressful) work situations and a motivation to participate in conflict management. A few participants having an executive position described that they changed their management style as a result of the workshop.

##### Changed perception and handling of (stressful) work situations

Some Participants reported that they changed their perception of work situations, particularly in case of dilemma situations that might pose a significant reason for stress during the daily work routine:

*»Because (the dilemma*,* M.S.) was introduced as an official problem*,* it became easier… more normal. And it helps to make decisions. We actually have to make a lot of decisions. And because you recognise dilemmas*,* it is not so important how you decide*,* because you know this way or that way*,* both solutions are unpleasant. I will go this way and keep working and I can work faster that way. And reduce my stress.« (Physician)*

In that way, participants valued the explanation that they cannot fulfil all work requirements at any time and will potentially disappoint colleagues or patients in situations of heavy workloads. They further achieved a better understanding about ways and principles to prioritize their (work) assignments:

*»That is quite helpful and it is something I always do for myself*,* to say stop*,* what kind of demands can you meet*,* who loose and what is the best option for me? How does that make me feel best or how can I best justify it?« (Nurse)*

Many participants reported that they considered their own needs somewhat stronger and for instance insisted on work breaks, lunchbreaks or punctual end of work, as a result of their changed perception and priority setting:

*»And meanwhile I am really aware that I cannot fulfil all requirements (…). I will go for lunch now. Or I will go home now because it is six pm and we will work off the rest tomorrow. That is somewhat easier for me now.« (Physician)*

##### Conflict management and problem solving

A few participants described that they were more motivated to become involved in problem solving as a result of the workshops:

*»That I could open myself in the group about problems I identified in the group. And I have learned that other feel the same even if they have not talked about it yet. And we actually developed a new work time model that is more equitably and less straining (…). And that changed a lot in my opinion. Particularly for me*,* I have just noticed that you can not only complain or be passive but if you are actively involved you will find other people who have a similar view and then you can transform the system together in some way.« (Physician)*

##### Modified management style

Two participants having an executive position reported about their changed communication style with the team. For instance, they strengthen communication with the employees and took personal characteristics of employees into account.

*»I simply could not imagine a more intensive contact with the employees*,* for example a personal conversation in between - I would have found that rather unprofessional. But that* (kind of social interaction, M.S.) *was rather encouraged there*,* to strengthen it and to do so and*,* yes*,* to a certain extent I developed different kind of management philosophy.« (Physician)*

### Mixed-methods integration

**Fig. 2:**
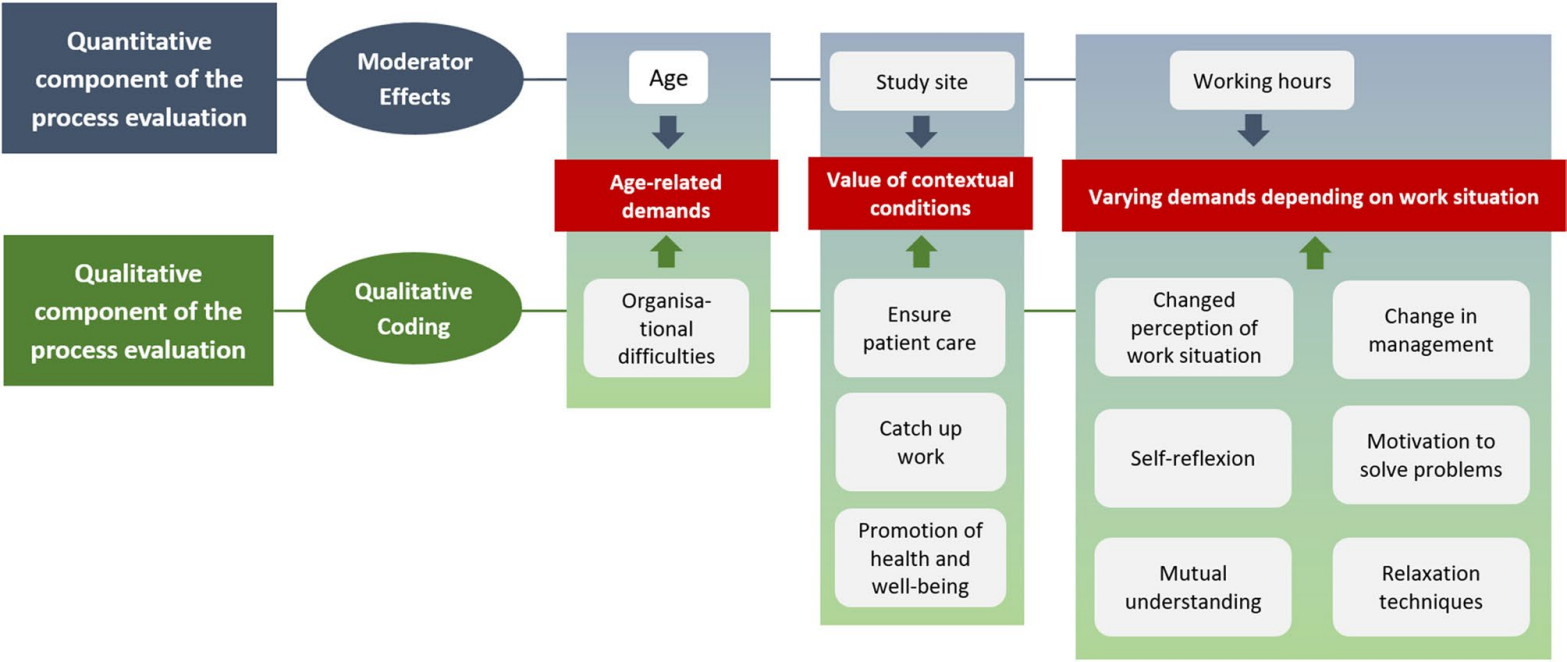
Integration of results of quantitative and qualitative study components

Figure 2 gives an overview of findings from the quantitative and qualitative components of the process evaluation.

The quantitative component of the study showed differences regarding the reduction of emotional and cognitive strain of participants associated to the intervention group, which can be complemented by qualitative results: For one thing, quantitative results showed varying emotional and cognitive strain with regard to participants’ age — while strain reduced in younger participants, it increased with advancing age of participants. This result indicates age-related demands of participants regarding the intervention. In that respect, qualitative results showed that participants valuated methods of intervention implementation differently. Due to the COVID-19 pandemic, intervention methods had to be adapted to ensure distance control by major parts of the intervention delivered online. Qualitative results showed that it was more difficult to adjust to such methods for older participants who, as a consequence, may have benefited less from the workshops.

Further, quantitative results showed that strain reduced at one study site only, while it remained stable at the other two, indicating that contextual conditions may have influenced the impact of the intervention. The relevance of contextual conditions was affirmed by qualitative results, i.e. participants reported varying conditions for workshop attendance due to working conditions and structural circumstances. Withdrawal from workshops often occurred to guarantee patient care, and to compensate for of short-term staff shortages or changed working schedules.

Further, quantitative results showed a reduction of strain in participants working full time as compared to participants working part-time, indicating varying demands of participants depending on the respective work situation. As part of the qualitative component, participants described a number of ways how they experience work related stress and methods used in regards to stress reduction. Findings show that adjustments of participants relate primarily to the individual level: Participants described particularly behaviour changes such as relaxation techniques and changed perception of (stressful) work situations (Fig. 2).

## Discussion

This mixed-methods process evaluation of a complex intervention to improve mental health of hospital staff showed that the effect of the intervention was moderated by some staff- and work-related variables, and mediated by fidelity to the intervention. Given the overall lack of effect of the intervention [30], it is hardly surprising that only few of the tested moderators (8 out of 60 possible) had an effect. Further, number of moderator effects decreased over time, which is not surprising in the light of the lack of long-term effectiveness of the intervention. Nonetheless, the effect of the intervention differed substantially, mostly by sociodemographic variables (gender, age), and to some extent also by work-related variables (working full- vs. part-time) and wider context (working at one of the three hospitals). This corresponds to findings of a recent systematic review which found a prominent role of socioeconomic status and work situation as moderators of the effectiveness of interventions to reduce stress in hospital employees [8]. Also in line with this review is our finding that the effect of the intervention is mediated by fidelity, indicating that it is essential to deliver an intervention as intended [25]. However, the finding that self-efficacy does not act as a mediator is not in line with a number of other studies in the field [18–22]. However, a closer look at the cited studies reveals that comparability is limited in many respects, including differences in participants, measures of self-efficacy and outcome, study designs, and methods to test mediation. E.g. some studies included only one group of health professionals (e.g. nurses in [18]), had “harder” outcomes than occupational stress (mental health in [18, 20]), and applied mere cross-sectional designs, limiting inference of causality [19–22].

Qualitative results confirmed and supplemented quantitative findings. Despite the lack of proof of efficacy of the overall intervention, participants of all professional groups and hierarchical levels mostly described the organisation and content alignment as very well thought out and performed. However, workshop attendance of participants as well as participants’ adjustments regarding stress reduction was limited due to the existing working structures. Participants’ adjustments regarding stress reduction related primarily to the individual level, in particular behaviour changes and changed perception of (stressful) work situations. Therefore, results suggest that psychosocial and cognitive interventions can contribute to behavioural changes, particularly when focused on individual coping strategies and workplace communication. Contrarily, workshop elements were assessed as less helpful if recommendations for action referred to hospital structures. Our results also point to the importance of extensive support for mental health promotion by the hospital management as well as sufficient resources to attain a lasting implementation of interventions. Further, qualitative findings revealed some aspects emphasising the importance of working conditions and organisational support (e.g. not be able to turn off the phone, being always-on, no break), pointing to the need for organisational interventions to address the causes of stress, e.g. by changing work schedules, improving communication, and enhancing job control [47]. Our results showed that contextual tailoring was appreciated and led to better participation and outcomes. Further, the positive feedback towards cross-disciplinary workshops suggests that future interventions should emphasise mutual understanding and team cohesion. While digital delivery had mixed reviews, particularly among older participants, it proved feasible during the COVID-19 pandemic and can offer scalability and flexibility with appropriate participant support.

This study has a number of limitations. First, the SEEGEN study was powered for testing effectiveness, not for complex analyses in a process evaluation. Thus, the low number and magnitude of moderator and mediator effects might also be due to lack of power and low sample size, problems most post-hoc process evaluations face. However, reporting interaction effects may elucidate important trends which may point to meaningful differences if replicated across studies [10]. Second, also due to challenges posed to the research teams and study participants by the COVID-19 pandemic, data collection was cumbersome, resulting in a substantial amount of missing data, e.g. regarding uptake of the intervention. Third, we used a simple approach to analyse quantitative data (linear models). Results may change if more sophisticated methods were used (e.g. multilevel models). Fourth, while the analyses of moderators and mediators included important variables usually investigated in process evaluations (socioeconomic status for moderation, fidelity and uptake of the intervention for mediation), analyses were post-hoc, lacking a concise conceptual framework. We might have also missed important effects, e.g. unobserved workplace dynamics which might influence the intervention’s effectiveness. Fifth, participants of the qualitative study represented different hierarchical levels, professional groups and hospital sites. A broad selection of participants generally enables to cover a diversity of opinions. However, professional and site affiliations were unevenly distributed so that some groups of participants were represented stronger than others. Sixth, employee participation has not been explicitly incorporated into the design of the intervention, which is important, especially to adequately address organisational change [48].

### Conclusions

The high complexity of the intervention with different modules and target groups hindered implementation and effectiveness. Taken together, results of the moderator analysis do not allow the intervention to be tailored to subgroups. However, mediator analysis showed that although modules varied across groups of participants and participants chose different modules, several factors influencing implementation of the intervention were similar. To improve implementation taking these barriers and facilitators into account seems crucial, as well as the further implementation and evaluation of smaller components of the intervention, added by theory development and refinement.

#### Implications for practice and policy recommendations

Our results indicate various implications in order to improve interventions to reduce work-related stress in hospital staff. Interventions should be less complex and more target-oriented, by addressing certain professional groups with a focus on their specific working conditions on site. Therefore, interventions that account for local, contextual factors, such as staffing levels, shift models, and departmental structures, might be more likely to be feasible and impactful. In addition, our results highlight the importance of hospital management’s commitment to providing comprehensive support for mental health promotion measures in order to achieve sustainable implementation of interventions. Future interventions may only be worthwhile if staff wellbeing is institutionally prioritised, clearly supported by leadership, and integrated into hospital policy.

## Supplementary Information

Supplementary Material 1.

## Data Availability

Quantitative data cannot be shared because participants did not give consent to sharing their data. All transcript fragments which informed the analysis of qualitative data presented in this publication are included within the paper and its online supplement files. Full transcripts are not publicly available due to their containing information that could compromise the privacy of research participants.

## Abbreviations

SEEGEN: Seelische Gesundheit am Arbeitsplatz Krankenhaus [Mental Health in the Workplace hospital]; study acronym
RCT: Randomised controlled trial
IRR: Irritation Scale
WHO-5: World Health Organization Well-Being Index
PSC-12: Psychosocial Safety Climate Scale
SOSES: Occupational Self-Efficacy Scale
