# Supplementary material for: Improving mental health and well-being of hospital staff: mixed-methods process evaluation of the SEEGEN trial
Source: BMC Public Health. 2025 Aug 16;25:2811. doi: 10.1186/s12889-025-24101-4 (PMC12357457; doi:10.1186/s12889-025-24101-4)
Supplement: Supplementary file 1 — Supplementary Material 1. [file 12889_2025_24101_MOESM1_ESM.docx]

**Improving mental health and well-being of hospital staff: mixed-methods process evaluation of the SEEGEN trial**

**Online supplement**

Table S1: SEEGEN Fidelity Scale

| 1. I learned a lot of things through the program that were useful to me. |
| --- |
| 1. I understood the most important contents well. |
| 1. The structure (red thread) of the intervention was always comprehensible. |
| 1. I was encouraged to think and critically evaluate the topic. |
| 1. The topic of the offer is very relevant to my current professional activity. |
| 1. I feel able to implement the content of the course in my everyday work. |
| 1. In my day-to-day work I have sufficient opportunity to put the content of the course into practice. |
| 1. I have already applied content from the offer in practice. |
| 1. The trainer seemed competent and well prepared for the offer. |
| 1. The trainer responded to questions and suggestions from the participants. |
| 1. There was a very pleasant working atmosphere. |
| 1. The trainer provided ample opportunity for practical exercises and references to everyday life. |
| 1. The offer encouraged my interest in the topic. |
| 1. Participation in the offer was very worthwhile. |
| 1. The general conditions (room, catering, etc.) on site were suitable. |
| 1. I would recommend participation in this program to others. |
| 1. What final grade would you give the event? (1=very good - 6=insufficient) |

Notes. Value labels items 1-16: not applicable (0); rather not applicable (1); partially applicable (2); rather applicable (3); fully applicable (4). Item 17 recoded: 5 and 6 to 0; 4 to 1; 3 to 2; 2 to 1; 1 to 0.

Table S2: Interview guide

| Theme | Impulse | Further Enquiry | Background |
| --- | --- | --- | --- |
| Introductory questions:   1. At first, I would like to ask you what kind of job position you have and which workshop you took part in. 2. Have you also participated in a round-table or booster-session? | | | |
| Organizational issues | How do you feel about the organisation of the workshop(s) you attended? | How were you informed about the SEEGEN workshops and the project?  How were you able to reconcile participation in the workshop with your everyday work?  What kind of difficulties may arise from participating in a workshop?  What did you especially like about the organisation?  How could things be better organised?  *Can you give an example?*  *Can you explain this in more detail?* | Opportunities for participation  Barriers  Workplace situation |
| Content issues | How do you feel about the content of the workshop(s) you attended? | In your opinion: What topics were especially helpful to reduce negative stress?  In your opinion: Which topics were perhaps less helpful?  What did you particularly like about the content? What did you may not like about the content?  *Can you give an example?*  *Can you explain this in more detail?* | Did the workshops addressed reasons for negative stress at the workplace?  Differences between professional groups or hierarchy levels? |
| Implementation / applicability | After your workshop attendance:  What has changed after the workshops or participation in the workshops? | (personal):  How could you implement the elements of the workshop?  *If not already raised*: To what extent has it helped to reduce negative stress?  What content would you like to realise?  For what reasons can you fail when implementing content?  (institutional):  What has changed in your department after the workshops?  *If not already raised*: To what extent have the changes in the department led to a reduction in negative stress? How did the process of change go in your department?  What could be the reasons why no changes have taken place in your department?  *Can you give an example?*  *Can you explain this in more detail?* | Workshops generate stimuli to change work situation  Differences between professional groups or hierarchy levels?  Realisation personal/institutional |
| Closing | Are there any other aspects of the SEEGEN workshops that you think are important that we haven't talked about yet?  What is your personal conclusion on the SEEGEN project? | | |

Table S3 Code development

| **Work steps**  *Differentiation of Codes* |  | | | |
| --- | --- | --- | --- | --- |
| 1. Deductive | **Organisation** | | **Workshops** | **implementation / Applicability** |
| 2. Inductive  ***Main Themes*** | **Hospital sites** | **Project/Workshop** | **Workshops – content-related aspects** | **Implementation and knowledge transfer** |
| 3. Inductive | - Opportunities for participation - Promotion of health and wellbeing | - Positive and critical aspects - Changes in consequence of Covid-19 - Round tables - Groups of participants | - Style of presentation - Extent/Structure - Atmosphere - Content - Subjective perception of stress/dealing with stress | - Acquired knowledge, utilized Barriers - absent of trust in management staff - structural changes unknown - No / very low changes - Changes affecting the workplace - Dissemination of workshop content - Reduced strains on the job in consequence of Covid-19 - Round tables |
| 4. Inductive | Differentiation: Code: Opportunities for participation in *2 subcodes:* (i) Barriers for participation in clinical context; (ii) Paid leave. | Recoding: Code: positive and critical aspects in *5 subcodes:* (i) Early announcement; (ii) Sufficient information; (iii) Short distances to the venue; (iv) Accessibility of project staff; (v) Unclear responsibilities of project staff. | Differentiation: Code: Content in *8 Subcodes*: (i) Theoretical models reflected; (ii) Deepening practical exercises; (iii) Addressed course participants causes of stress; (iv) Addressed common causes of stress; (v) Conveyed comprehensibility; (vi) Follows up on already-existing knowledge; (vii) Currently unknown; (viii) Not considered helpful. | Differentiation: Code: Acquired knowledge, utilized in *9 subcodes*: (i) Amended approach/work priority; (ii) Amended leadership behaviour; (iii) Self-reflection; (iv) Incentive to address the problem; (v) Mutual understanding; (vi) Reassessment/changed perception; (vii) Relaxation technique; (viii) Perceiving and considering own needs; (x) Time out.  Differentiation: Code: Barriers in *3 subcodes*: (i) Little support from project staff; (ii) Work conditions; (iii) Additional workload in consequence of Covid-19 |
| 5. Inductive | Differentiation: Code: Barriers for participation in clinical context in *3 Subcodes:* (i) Catch up work; (ii) Comply with working schedule; (iii) Ensure patient care. |  |  | Differentiation: Code: work conditions in *6 Subcodes:* (i) Structural requirements; (ii) Missing culture of criticism; (iii) Time pressure; (iv) Team/colleagues; (v) Staff turnover; (vi) Ensure business operation/patient care.  Differentiation: Code: Structural requirements in 4 *Subcodes: (i)* communication; (ii) Staff shortage; (iii) Change resistant system; (iv) Premises. |

Table S4: Descriptives of outcome and process measures over time

|  |  | **T0** | **T1** | **T2** |
| --- | --- | --- | --- | --- |
| IRR  *mean (sd), n* | IG | 26.03 (9.49), 193 | 26.07 (10.10), 127 | 26.41 (10.03), 111 |
|  | CG | 26.17 (9.64), 208 | 25.26 (9.18), 175 | 26.46 (9.86), 160 |
| WHO  *mean (sd), n* | IG | 55.09 (18.85), 193 | 56.72 (21.92), 127 | 52.62 (20.85), 110 |
|  | CG | 58.00 (18.42), 208 | 55.49 (20.22), 178 | 52.41 (21.65), 160 |
| PSC-12  *mean (sd), n* | IG | 28.57 (9.68), 188 | 28.24 (10.30), 126 | 28.52 (10.27), 110 |
|  | CG | 29.47 (9.53), 205 | 30.43 (8.72), 174 | 28.83 (8.96), 157 |
| SOSES  *mean (sd), n* | IG | 4.41 (0.90), 181 | 4.40 (0.97), 120 | 4.44 (0.89), 104 |
|  | CG | 4.51 (0.85), 192 | 4.60 (0.76), 163 | 4.57 (0.82), 148 |
